# Metagenomics reveals biogeochemical processes carried out by sediment microbial communities in a shallow eutrophic freshwater lake

**DOI:** 10.3389/fmicb.2022.1112669

**Published:** 2023-01-11

**Authors:** Bo Kuang, Rong Xiao, Yanping Hu, Yaping Wang, Ling Zhang, Zhuoqun Wei, Junhong Bai, Kegang Zhang, Jacquelinne J. Acuña, Milko A. Jorquera, Wenbin Pan

**Affiliations:** ^1^College of Environment and Safety Engineering, Fuzhou University, Fuzhou, China; ^2^State Key Laboratory of Water Environment Simulation, School of Environment, Beijing Normal University, Beijing, China; ^3^Department of Environmental Science and Engineering, North China Electric Power University, Baoding, China; ^4^Department of Chemical Sciences and Natural Resources, University of La Frontera, Temuco, Chile

**Keywords:** metagenomics, sediment, microbial community, biogeochemical processes, Baiyangdian

## Abstract

**Introduction:**

As the largest shallow freshwater lake in the North China Plain, Baiyangdian lake is essential for maintaining ecosystem functioning in this highly populated region. Sediments are considered to record the impacts of human activities.

**Methods:**

The abundance, diversity and metabolic pathways of microbial communities in sediments were studied by metagenomic approach to reveal patterns and mechanism of C, N, P and S cycling under the threat of lake eutrophication.

**Results:**

Many genera, with plural genes encoding key enzymes involved in genes, belonging to Proteobacteria and Actinobacteria which were the most main phylum in bacterial community of Baiyangdian sediment were involved in C, N, S, P cycling processes, such as *Nocardioides* (Actinobacteria), *Thiobacillus*, *Nitrosomonas*, *Rhodoplanes* and *Sulfuricaulis* (Proteobacteria).For instance, the abundance of *Nocardioides* were positively correlated to TN, EC, SOC and N/P ratio in pathways of phytase, regulation of phosphate starvation, dissimilatory sulfate reduction and oxidation, assimilatory sulfate reduction, assimilatory nitrate reduction and reductive tricarboxylic acid (rTCA) cycle. Many key genes in C, N, P, S cycling were closely related to the reductive citrate cycle. A complete while weaker sulfur cycle between SO_4_^2−^ and HS^−^ might occur in Baiyangdian lake sediments compared to C fixation and N cycling. In addition, dissimilatory nitrate reduction to ammonia was determined to co-occur with denitrification. Methanogenesis was the main pathway of methane metabolism and the reductive citrate cycle was accounted for the highest proportion of C fixation processes. The abundance of pathways of assimilatory nitrate reduction, denitrification and dissimilatory nitrate reduction of nitrogen cycling in sediments with higher TN content was higher than those with lower TN content. Besides, *Nocardioides* with plural genes encoding key enzymes involved in *nasAB* and *nirBD* gene were involved in these pathways.

**Discussion:**

*Nocardioides* involved in the processes of assimilatory nitrate reduction, denitrification and dissimilatory nitrate reduction of nitrogen cycling may have important effects on nitrogen transformation.

## 1. Introduction

As an important component of global aquatic ecosystems, lakes only account for a small fraction (2.8%) of the land surface (Downing et al., 2006), however play a unique and essential role in biogeochemical cycles due to their possession of diverse microbes, and capability to hasten the nutrients cycling (Hakulinen et al., 2005; Liu et al., 2009; Song et al., 2012; Small et al., 2014; Verpoorter et al., 2014; Li et al., 2015; Huang and Jiang, 2016; Frade et al., 2020). Microorganisms are monitors to variation in the external environment in the lake and may be one of the most sensitive indicators (Kuang et al., 2022). Metabolism of organic and inorganic elements can be changed by microbial communities which impact biogeochemical environments of lake sediments (Song et al., 2012). Moreover, it is reported that the carbon cycle is dominated by the balance between carbon-fixing and carbon-consuming occurred in microorganisms; microbial nitrogen-transforming networks both attenuate and exacerbate human-induced global change; microbial P mineralization is a side-effect of microbial C acquisition and can be driven by it; the remineralization of the organic matter of seafloor is facilitated by sulfate-reducing microorganisms (Spohn and Kuzyakov, 2013; Bowles et al., 2014; Gougoulias et al., 2014; Kuypers et al., 2018). Such natural diverse conditions harbor hotspots of microbes.

Increasing attention have been paid to the environmental regulatory factors leading to eutrophication and changing the constitute of sediment bacterial community, such as pH, N, P, N:P ratio (N/P) and soil organic carbon (SOC; Howarth et al., 2011; Wen et al., 2012; Yi et al., 2021; Kuang et al., 2022). And overdosing or excessive enrichment of nutrients lead to eutrophication. In the past half century, human activities have greatly accelerated the flow of nutrients to lake ecosystem, resulting in extensive eutrophication (Rabalais, 2002). In many industrialized countries, although the input of P drops sharply due to the improvement of wastewater treatment plants, the N pollution is still high (Howarth et al., 2011). In sediments, the decay of algal biomass generates organic carbon and consumes oxygen, which is conducive to N loss through denitrification. Under natural conditions and control experiments, when algae gather, N loss will be reduced (Zhu et al., 2020). Eutrophication may produce positive feedback to increase phosphorus supply. For example, P storage in many sediments decreases and P flux into water increases (Bol et al., 2018). In addition, in sediment, with the progress of eutrophication, the increase of C input and the decrease of oxygen input, sulfide will accumulate faster (Corredor et al., 1999). Even when the organic carbon (OC) content is low, sulfate reduction also accounts for a lot. When the OC concentration is high, the sulfate reduction contribution is higher (Howarth, 1984). Relationship between N, P and OC are also reported that the loss of N and the increase of P leads to lower N/P but higher trophic level index (calculated by Chl-a, Secchi disk transparency and TP). Specifically, in eutrophic lakes, the enrichment of total organic carbon (TOC) and all forms of P in sediment can promote potential denitrification rate and eventually leads to N loss by regulating community composition and increasing the abundance of *nirS* denitrifiers (Zhang et al., 2018). The eutrophication of surface water is an endemic global problem, and the nutrient load from agriculture is an underlying and persistent driving factor (Withers et al., 2014). Pesticides containing organophosphorus (OPPs) which were widely used in agriculture for their insecticidal properties are one of the most commonly used chemical pesticides in China (Wu et al., 2009). It is common for OPPs to leave residues in ecosystems due to their indiscriminate application (Montuori et al., 2016; Liu et al., 2019). To the best of our knowledge, there are various levels of toxicity associated with the acute and chronic exposure of humans, animals, plants, and insects to OPPs (Sidhu et al., 2019). It is demonstrated that pesticide pollution exists in agricultural ecosystems. For example, in sediments of Sundays and Swartkops Estuary, the highest concentration of OPPs is, respectively, up to 8.07 μg/kg and 13.6 μg/kg (Olisah et al., 2021a,b). The concentration of parathion and paraoxon-methyl (the degradation product of parathion-methyl) which are the most common OPP pollutants in Tai Lake varies from 5.88 to 506 ng/l (Wei et al., 2022).

In shallow lakes, eutrophication is widespread which leads to phytoplankton and algal blooms and then hypoxic or anoxic episodes when the organisms submerge and decompose, and is generally caused by increased P, N and organic matter (Gao et al., 2021; Li et al., 2016). As the largest shallow freshwater lake in the North China Plain, Baiyangdian lake is essential for maintaining ecosystem functioning in this highly populated region (Han et al., 2020). However, increased anthropogenic activities in recent decades have gradually increased the amounts of nutrients discharged into the lake in domestic sewage and industrial wastewater from the Baoding City in the upper reaches via the Fu River. These discharges and nutrients transported from farmland and residential areas have caused nutrient enrichment, resulting in eutrophication of Baiyangdian lake (Zhu et al., 2019). And then, the activities of C, N, P and S cycling in Baiyangdian lake have changed. A vast and uncultured diversity of microorganisms is exposed by molecular genetic techniques, so that there is a huge breakthrough of environmental microbiology (Handelsman, 2004). This is the main advantage of metagenomics, because cultivating new microorganisms is a difficult technology. Massive sequence data generated by metagenome technology allows linking microbial communities with their biogeochemical functions and provides insights about abiotic and biological interactions of microbial communities (Grossart et al., 2020). However, there are a few studies on the influence of environmental factors on the biogeochemistry cycle of shallow lakes under the condition of eutrophication. In this study, we (1) characterize the microbial community structure in the sediments of Baiyangdian, and (2) reveal the effect of environmental factors in controlling microbiome composition of sediment, and (3) show the relationship between relative abundance of important taxa and functional genes for regulating C, N, P and S cycling, as well as their metabolism pathway for further quantifying bacterial functional potential in biogeochemical cycling processes in inland freshwater lake floors under eutrophic threats.

## 2. Materials and methods

### 2.1. Study area and sample collection

**Figure 1:**
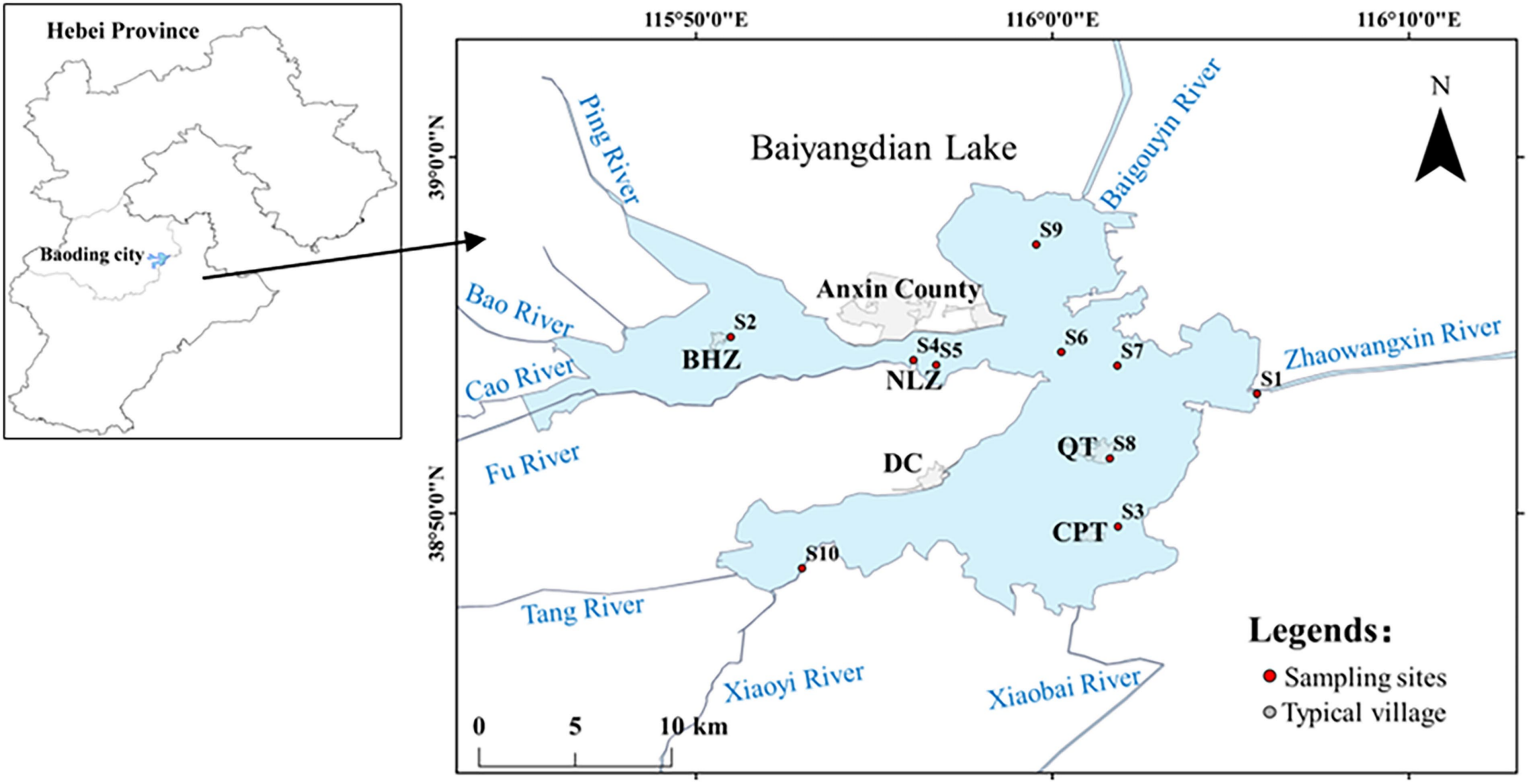
Sampling sites of Baiyangdian Lake.

In northern China, Baiyangdian Lake is located in the center of Hebei province at 38°43′ to 39°02′ N, 115°38′ to 116°07′ E (Figure 1) with a watershed area of 781 km^2^. Baiyangdian is the ecological hinterland of Xiong’an New Area. As a carrier of ecological resources and a sink of environmental load, water environment of Baiyangdian is tightly related to the ecological security of Xiong’an New Area. In our study, we want to basically reflect the whole physicochemical property of Baiyangdian Lake, so total 10 typical sediment samples (0–10 cm depth) which were intensively mixed of 4–6 buckets with grab bucket were collected during November 25 ~ 26, 2021. Specific sampling sites were marked as S1 to S10 in Figure 1. S1, S2, S3 and S8 are typical human gathering areas locating near the village. S6 and S7 are located near the inner river. S9 is located in open water and S10 nears to entrance of lake. S4 and S5 are near to the Anxin County. The villages of Beihezhuang (BHZ), Nanliuzhuang (NLZ), Duancun (DC), Quantou (QT) and Caiputai (CPT) were also marked on Figure 1. The samples were transported to the lab on ice and were kept at 4°C and −20°C, respectively, for further analysis.

### 2.2. Determination of physicochemical property and organophosphorus pesticides

Sediment samples were air-dried, crushed, and passed through meshes of 0.25 mm or 2 mm during laboratory analysis. Electrical conductivity (EC) and pH of sediment were measured at a soil to water mass ratio of 1:5 using a surveying instrument (P16 pH/EC/DO determinator, Yoke instrument, shanghai, China) equipped with a calibrated combined glass electrode (Kuang et al., 2022). SOC was determined using wet oxidation with K_2_Cr_2_O_7_; total nitrogen (TN) concentration was measured using the classical Kjeldahl digestion method and the total phosphorus (TP) concentration was determined by alkaline digestion followed by molybdate colorimetry (Liu et al., 2013).

Eight OPPs (diazinon, parathion-methyl, fenitrothion, malathion, chlorpyrifos, fenthion, quinalphos, ethion) were measured. Briefly, 10 g of sediment after being centrifuged was extracted by 10 ml acetonitrile. After shaking by hand for 30 s, 7.5 g anhydrous MgSO_4_ and 1 g NaCl were added. Subsequently, the mixture was centrifuged at 5000 rpm for 5 min after shaking for 30 s. The extract (1.8 ml) was mixed with 100 mg primary secondary amine sorbent, 150 mg anhydrous MgSO_4_ and 30 mg graphitized carbon black. They were shaken vigorously for 30 s and centrifuged at 9000 rpm for 5 min (Olisah et al., 2021b). The final extracts were used to analyze the OPPs by Gas Chromatography–Mass Spectrometry (GCMS-QP2020 NX, Shimadzu, Japan) fitted with SH-Rxi-5Sil MS column (30 m × 250 μm × 250 μm), with 2 μl volume being injected automatically. The split less mode was applied for injection and the injector inlet temperature was 250°C. The column temperature was programmed as follows: from 90°C to 180°C for 1 min at 25°C/min, from 180°C to 270°C for 1 min at 3°C/min, from 270°C to 310°C at 20°C/min for 3 min, given a total run time of about 41 min. Helium (99.9%) was used as the carrier gas at a constant flow rate of 1.0 ml/min, and nitrogen was the make-up gas. Full-scan analysis (50–450 m/z) was used to determine cleanup effects, and selected ion monitoring mode was used for measure.

### 2.3. DNA extraction and metagenomic sequencing

Total genomic DNA of sediment were extracted using ZNA^®^ Bacterial DNA Kit for Soil (Omega Biotech, United States) according to the manufacture’s instruction. The fresh sediment sample was used directly for DNA extraction. Extracted DNA was checked using 2% agarose gels. After quality assessment, DNA was stored at −80°C until further analyses. Metagenomic libraries of ten sediment samples were performed by Illumina HiSeq 2,500 platform. Raw sequence data (40.37 Gb) were generated. Sequences were cleaned and assembled using Seqprep, Sickle, BWA, and SOAPdenovo (Version 1.06), and the length of contigs >500 bp were retained for further bioinformatics analyses. CD-HIT was used to build a non-redundant gene catalog, and MetaGene software was used for ORF prediction. Taxonomic assignment was carried by using BLASTP alignment against the integrated non-redundant (NR) database of the National Center for Biotechnology Information (NCBI; Tatusov et al., 2003). In addition, the resulting genes were annotated and classified in species and function. Raw reads were uploaded to the NCBI database under BioProject PRJNA908081.

### 2.4. Bioinformatics analysis

On one hand, to carry out composition annotation, Unigenes were blasted to sequences of bacteria, eukaryota, archaea and viruses extracted from the NR database (Version 20,200,604) of NCBI using DIAMOND software (Version 0.8.35). On the other hand, for function analysis, DIAMOND software was adopted to blast Unigenes to KEGG (Kyoto Encyclopedia of Genes and Genomes) database (Version 94.2; Kanehisa et al., 2014; Buchfink et al., 2015). Then, genes encoding key enzymes involved in C fixation, N, S and P cycling were identified according to KEGG. Moreover, correlation analysis, calculated by IBM SPSS Statistics (Version 26), was used to investigate the relationships between environmental parameters and different functional profiles such as C, N, P, S cycling. Additionally, the dominant metabolic type of different functions was also determined by DIAMOND.

## 3. Results

### 3.1. Physicochemical property and organophosphorus pesticides

As shown in Table 1, the pH of sediment varied from 7.78 to 8.35 reflecting the slightly alkaline in the Baiyangdian Lake; the average value of EC in sediment was 410 μS/cm. TN varied significantly across sample sites with average value around 2,400 (±809) mg/kg and TP varied with average value around 515 (±92) mg/kg. No significant correlation was found between TP, OPPs and pH, EC, TN, SOC, N/P in the sediments from Baiyangdian Lake (Supplementary Table S1). Among the eight OPPs, the detection rates of diazinon and fenitrothion in sediment reached 50 and 60%, respectively, and were higher than the rest of six pesticides. The detection rate of parathion-methyl and malathion in sediment were lower than those of diazinon and fenitrothion, while the concentrations were higher than those of diazinon and fenitrothion. The highest level of total OPPs was measured in site S10 reaching around 97 mg/kg (average value) and the lowest concentration of total OPPs was determined in the sits S6 as low as 3.2 mg/kg (average value). TN had significant negative correlation with pH (*p* < 0.05) and positive correlation with EC, SOC, N/P (*p* < 0.01). In addition, EC had significant negative correlation with pH (*p* < 0.05) and positive correlation with N/P (*p* < 0.01; Supplementary Table S1).

**Table 1:** Physicochemical properties of sediment samples from Baiyangdian Lake.

Sites	pH	TN (mg/kg)	TP (mg/kg)	EC (μS/cm)	SOC (%)	N/P	OPPs (μg/kg)
S1	8.35	1446.84 (152.96)^d^	436.67 (102.15)^ab^	230	1.33 (0.47)^bc^	3.31	4.38 (±6.19)^b^
S2	7.78	3320.52 (193.71)^ab^	572.22 (70.83)^ab^	419	1.38 (0.23)^bc^	5.8	15.47 (±1.11)^b^
S3	7.86	3634.56 (356.97)^a^	402.22 (24.66)^b^	559	3.34 (1.28)^a^	9.04	16.97 (±1.05)^b^
S4	7.83	2724.03 (262.19)^bc^	689.26 (145.01)^a^	547	2.9 (0.25)^ab^	3.95	79.94 (±40.88)^a^
S5	7.99	1613.51 (136.73)^d^	595.93 (68.24)^ab^	247	1.65 (0.44)^abc^	2.71	34.48 (±11.46)^b^
S6	8.16	2497.72 (60.22)^c^	513.7 (167.87)^ab^	270	2.7 (0.25)^abc^	4.86	3.2 (±1.54)^b^
S7	8.31	1631.05 (151.87)^d^	554.45 (126.35)^ab^	262	1.36 (0.3)^bc^	2.94	17.28 (±2.78)^b^
S8	8.06	1588.95 (235.26)^d^	355.93 (44.97)^b^	405	1.02 (0.29)^c^	4.46	14.6 (±7.45)^b^
S9	8.10	2064.39 (191.91)^cd^	524.07 (54.03)^ab^	383	1.31 (0.45)^bc^	3.94	9.29 (±6.54)^b^
S10	7.98	3481.93 (651.02)^a^	511.85 (125.7)^ab^	777	2.74 (1.62)^abc^	6.8	97.47 (±15)^a^

Values were presented by means (with standard deviation). Lower case letters-superscripts denote significant differences among the different groups within a column (*p* < 0.05).

### 3.2. Taxonomic profiles of bacterial community in sediment

**Figure 2:**
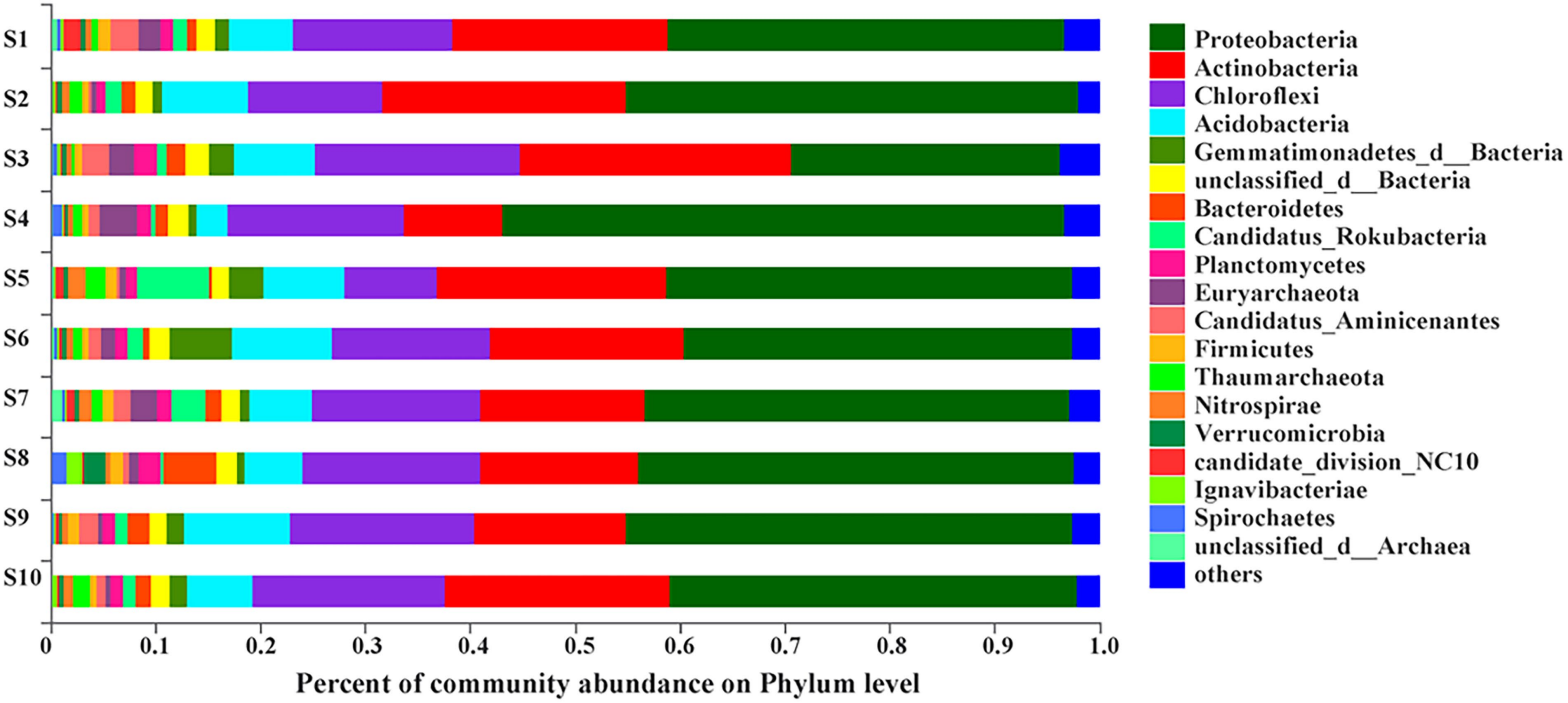
Bacterial communities’ composition in sediments at phylum level of Baiyangdian lake.

At the phylum level, more than 1% of total microbial community composition in each sediment sample was picked out to analyze species and abundance of bacterial community. The top eight phyla followed the order of Proteobacteria (25.64% ~ 53.63%), Actinobacteria (9.39% ~ 25.96%), Chloroflexi (8.83% ~ 19.45%), Acidobacteria (2.99% ~ 10.01%), Gemmatimonadetes (0.67% ~ 5.97%), unclassified_d__Bacteria (1.55% ~ 2.16%), Bacteroidetes (0.33% ~ 5.09%), Candidatus_Rokubacteria (0.33% ~ 6.93%; Figure 2). At site S3, Proteobacteria had the lowest relative abundance among all samples while Actinobacia and Chloroflexi had the highest. In addition, the sediment sample from site S4 had the highest relative abundance of Proteobacteria but the lowest relative abundance of Actinobacteria, Acidobacteria and Gemmatimonadetes. Candidatus_Rokubacteria had the highest relative abundance in S5 but lowest in S8. Bacteroidetes (opposite to Candidatus_Rokubacteria) had the highest relative abundance in S8 but lowest in S5. Chloroflexi was the most abundant in site S5. The relative abundance of Gemmatimonadetes and Acidobacteria were the highest in S6 and S9, respectively. As shown in Table 2, many genera were involved in C, N, S, P cycling processes, such as, *Thiobacillus*, *Nitrosomonas*, *Aromatoleum*, *Rhodoplanes*, *Sulfuricaulis*, *Pseudomonas*, *Desulfatitalea* and *Desulfobulbus* of Proteobacteria; *Nocardioides* of Actinobacteria; *Methanothrix* of Euryarchaeota. A few key genes involved in each cycle were showed in Supplementary Table S2. For example, *napAB, narGHI, nasAB, nifDHK, nirBD, nirKS, norBC* and *nrfAH* were involved in nitrogen cycling; *aprAB* was involved in phosphorus cycling. Many genera with plural genes encoding key enzymes involved in these genes. Such as *Thiobacillus*, *Nocardioides*, *Gaiella*, *Actinomadura*, *Thioalkalivibrio*, *Ramlibacter*. Then, at the phylum level, for the bacterial community in the sediments of Baiyangdian lake, the relative abundance of Chloroflexi had significant positive correlation with EC while the relative abundance of Candidatus_Rokubacteria had negative correlation with it.

**Table 2:** Major taxa involved in C, N, P, S cycling in Baiyangdian lake sediments based on KEGG annotation results of metagenomes.

Cycling processes	Metabolic pathways	Major taxa
Carbon fixation	*1: 3-HP/4-HB*	*Rhodoplanes, Syntrophus, Methylibium*
	*2: 3-HP*	*Nocardioides, Aestuariivirga, Sulfurisoma*
	*3: WL*	*Methanothrix, Desulfobacca, Desulfobulbus*
	*4: rTCA*	*Nocardioides, Thiobacillus, Anaeromyxobacter*
	*5: CBB*	*Thiobacillus, Sedimenticola, Sulfuricaulis*
Nitrogen cycling	*6: Nitrification*	*Nitrosomonas, Sulfurifustis, Streptomyces*
	*7: N fixation*	*Thiobacillus, Desulfobulbus, Geobacter*
	*8: Denitrification*	*Thiobacillus, Dechloromonas, Anaeromyxobacter*
	*9: Assimilatory nitrate reduction*	*Nocardioides, Thiobacillus, Dechloromonas*
	*10: DNRA*	*Thiobacillus, Nocardioides, Gaiella*
Sulfur cycling	*11: Sox system*	*Thiobacillus, Aromatoleum, Sulfuricaulis*
	*12: Dissimilatory sulfate reduction and oxidation*	*Thiobacillus, Sulfuricaulis, Nocardioides*
	*13: Assimilatory sulfate reduction*	*Nocardioides, Nitrososphaera, Thiobacillus*
Phosphorus cycling	*14: Regulation of phosphate starvation*	*Thiobacillus, Steroidobacter, Nocardioides*
	*15: Phosphorus*	*Thiobacillus, Nocardioides, Candidatus_Methanoperedens*
	*16: Inorganic phosphate solubilizing*	*Pseudomonas, Nocardioides, Thiobacillus*
	*17: Phosphonate degradation*	*Desulfatitalea, Thiobacillus, Candidatus_Methanoperedens*
	*18: Phytase*	*Solirubrobacter, Nocardioides, Kribbella*
	*19: Phosphoesterase*	*Nocardioides, Cryobacterium, Aromatoleum*

3-HP/4-HB, 3-hydroxypropionate/4-hydroxybutylate cycle; 3-HP, 3-hydroxypropionate bicycle; WL, Wood-Ljungdahl pathway; rTCA, reductive tricarboxylic acid cycle; CBB, Calvin–Benson–Bassham cycle; DNRA, dissimilatory nitrate reduction to ammonium.

### 3.3. Carbon fixation, nitrogen, sulfur, phosphorus cycling metabolism through metagenomic analysis

**Figure 3:**
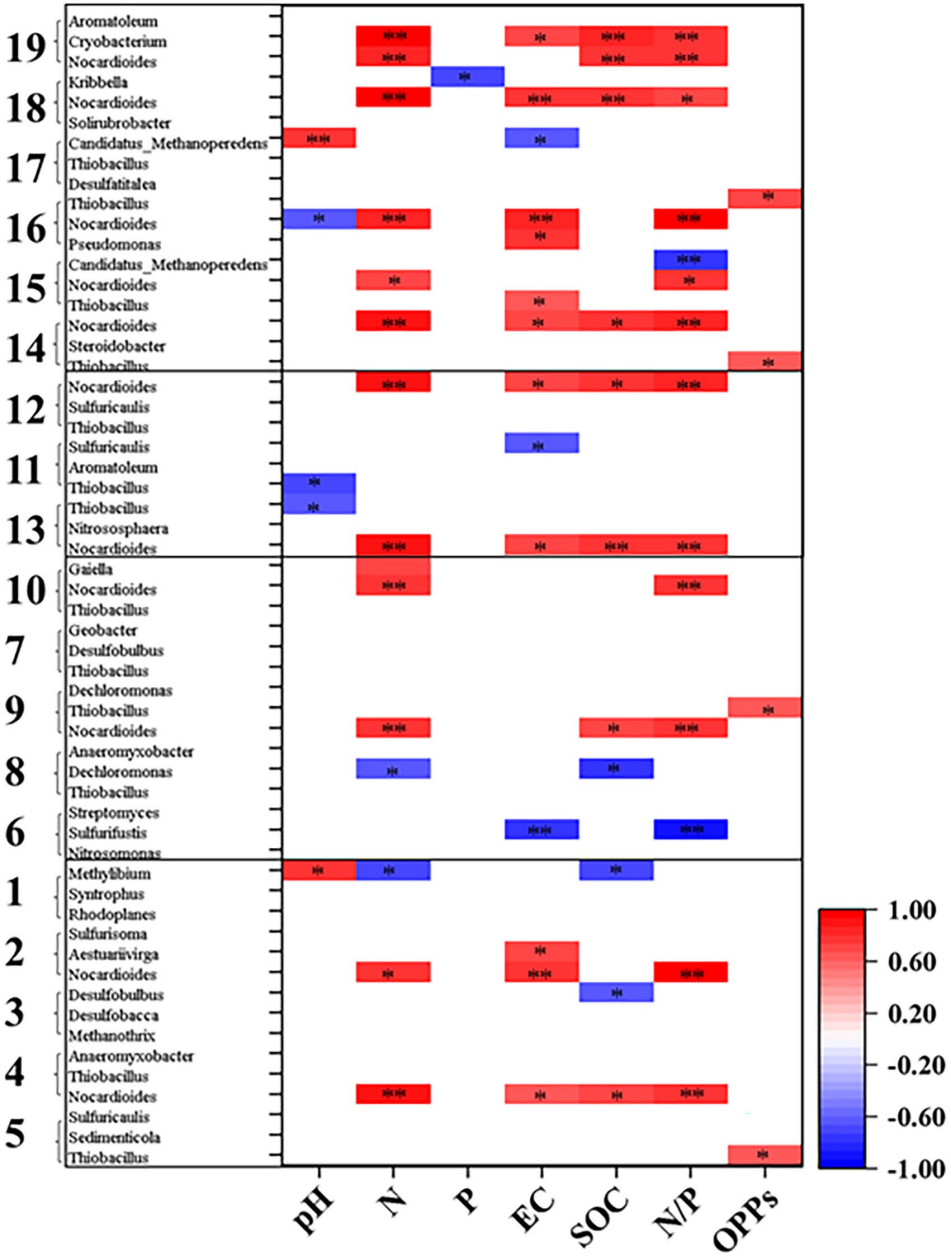
Spearman’s correlation coefficients between major taxa involved in C, N, P, S cycling and sediment properties. Colored squares indicate significant correlations at *p* < 0.05 (*) or *p* < 0.01 (**) level and blank space indicates no significant correlation. The digits of y-axis represent different pathways showed in Table 2.

**Figure 4:**
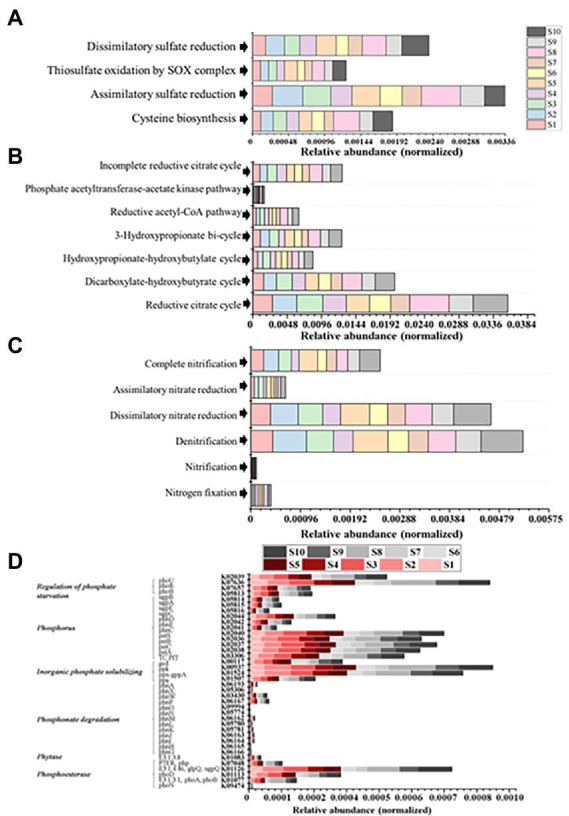
**(A)** Relative abundance of S metabolism modules. **(B)** Relative abundance of C fixation modules. **(C)** Relative abundance of N metabolism modules. **(D)** Relative abundances of genes encoding key enzymes involved in P cycling.

**Figure 5:**
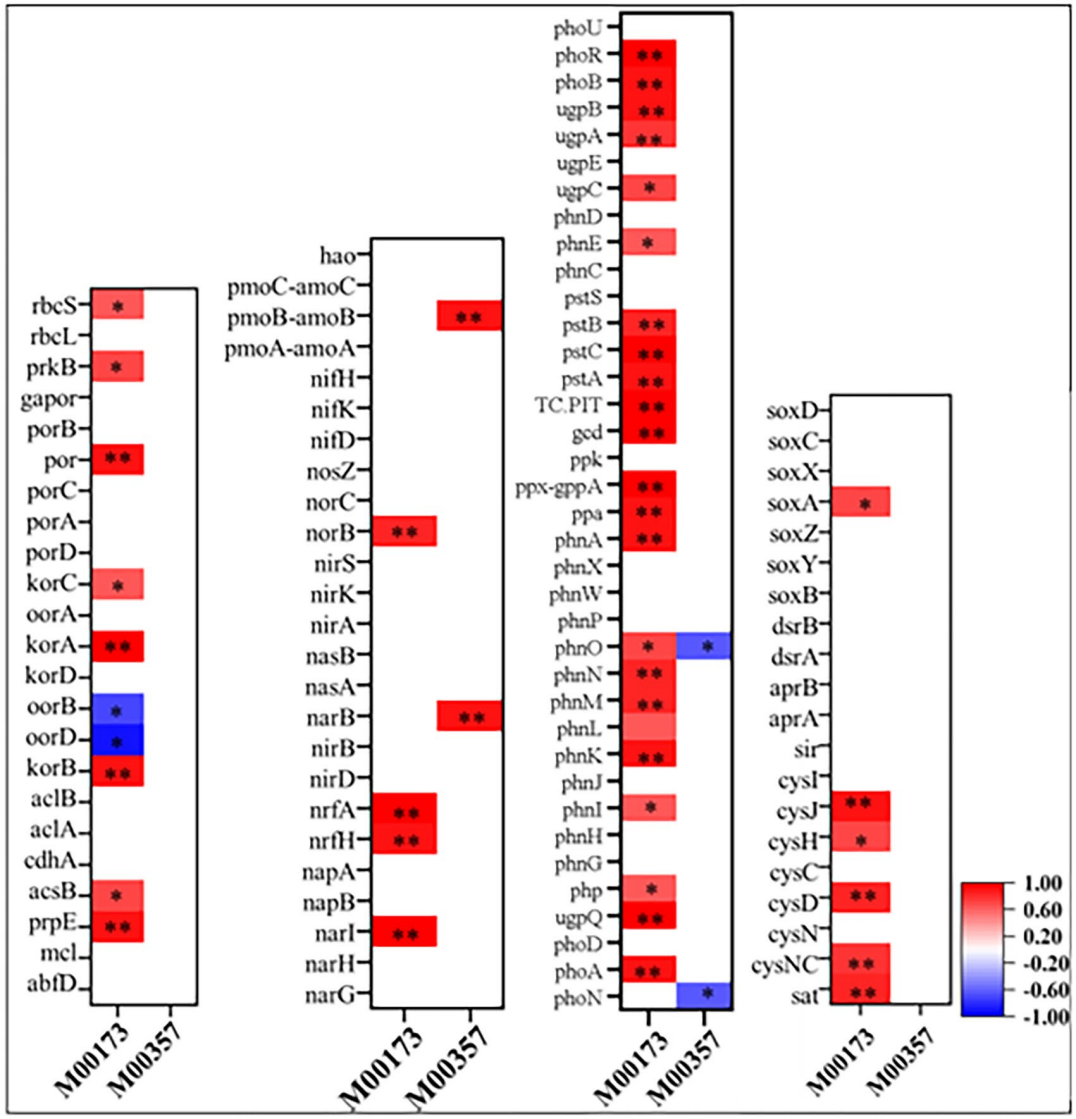
Spearman’s correlation coefficients between key genes in C, N, P, S cycling and modules of methane metabolism and carbon fixation. Colored squares indicated significant correlation at *p* < 0.05(*) or *p* < 0.01(**) level while blank space indicated unsignificant correlation between key genes abundance and the corresponding modules abundance showing below in the x-axis.

**Figure 6:**
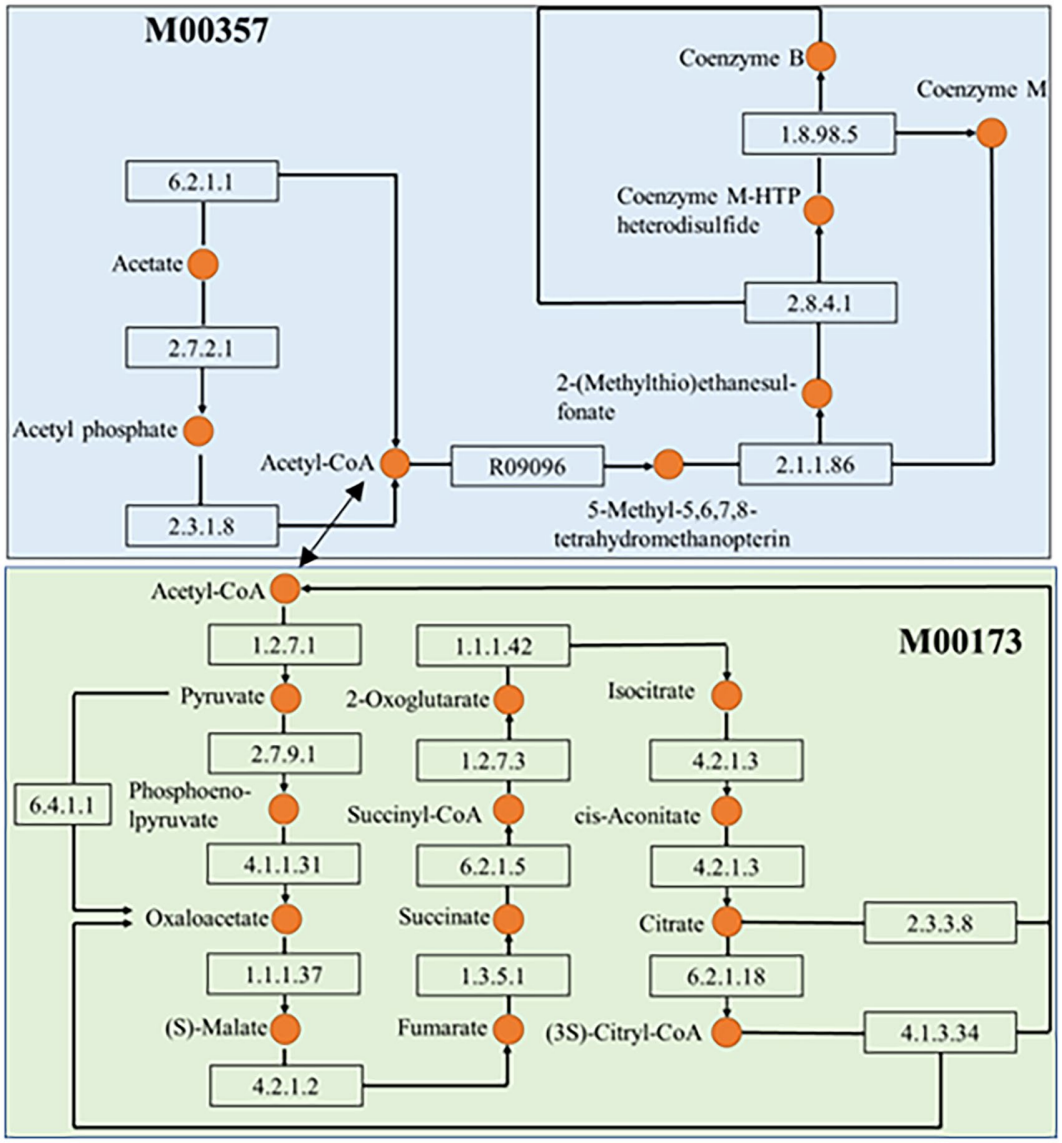
Interrelationship between the methanogenesis, acetate = >methane (M00357) in methane metabolism and the module reductive citrate cycle (Arnon-Buchanan cycle; M00173) in carbon fixation. The number in the rectangle represents the enzyme and the orange circle represents the substance noted on the left.

As shown in Supplementary Figure S1, at KEGG pathway level 2, global and overview maps were the dominant metabolic subsystems in the sediment system. Metabolism was also the indispensable function in the system of sediment, such as, metabolism of carbohydrate, amino acid, energy, cofactors and vitamins, lipid, nucleotide, xenobiotics, terpenoids and polyketides. In addition, carbohydrate metabolism, amino acid metabolism and energy metabolism had relative abundances of 10.47, 7.64, and 5.69%, respectively. Carbon fixation pathway, N, S cycling modules and genes encoding key enzymes involved in P cycling were analyzed based on the KEGG database. Major taxa involved in C, N, P, S cycling based on KEGG annotation results of metagenomes were shown in Table 2. Most of them belonged to Proteobacteria phylum. In addition, among the major taxa, the genus *Nocardioides* had played an important role in many metabolic pathways of C, N, P, S cycling and was affected by many environmental factors. As shown in Figure 3, for example, the abundance of *Kribbella* was negatively correlated with TP in pathway of phytase. The relative abundance of *Thiobacillus* was positively correlated with the concentration of OPPs in pathways of regulation of phosphate starvation, inorganic phosphate solubilizing, assimilatory nitrate reduction and CBB (Table 2). The abundance of *Nocardioides* were positively correlated to TN, EC, SOC and N/P in pathways of phytase, regulation of phosphate starvation, dissimilatory sulfate reduction and oxidation, assimilatory sulfate reduction, assimilatory nitrate reduction and rTCA. The abundance of *Candidatus_Methanoperedens* was positively related to pH (negatively related to EC) in pathway of phosphonate degradation and was negatively related N/P in pathway of phosphorus. Sulfur metabolism pathway was mainly composed of 4 reaction modules (Figure 4A): assimilatory sulfate reduction, dissimilatory sulfate reduction and oxidation, thiosulfate oxidation by SOX complex and cysteine biosynthesis; C fixation pathway was mainly composed of 7 reaction modules (Figure 4B): incomplete reductive citrate cycle, phosphate acetyltransferase-acetate kinase pathway, reductive acetyl-CoA pathway, 3-Hydroxypropionate, hydroxypropionate-hydroxybutylate, dicarboxylate-hydroxybutyrate and reductive citrate cycle; N metabolism was mainly composed of 6 reaction modules (Figure 4C): complete nitrification, assimilatory nitrate reduction, dissimilatory nitrate reduction, denitrification, nitrification and nitrogen fixation; and key genes of P cycling were divided into 6 types (Figure 4D). P-cycling genetic signatures in sediment metagenome involved in phosphoesterase, phytase, phosphonate degradation, inorganic phosphate solubilizing, phosphorus and regulation of phosphate starvation. For example, high abundance genes *phoURB* involved in regulating of phosphate starvation; *pstABC* involved in P transport; *glpQ* and *phoD* involved in phosphoesterase. Moreover, the highest proportion M00176 was the main pathway of S metabolism and the highest proportion M00377 was the main pathway of C fixation. In the N metabolism module M00377 occupying the highest proportion, nitrates were converted to nitrogen by denitrification. As shown in Figure 5, many key genes in C, N, P, S cycling were correlated with module M00357 and M00173. In these two modules (Figure 6), these genes were mainly involved in the module reductive citrate cycle of C fixation. In addition, the abundance of genes *corBD* were proved to be negatively correlated with M00173. The module methanogenesis in methane metabolism was positively correlated with abundance of genes *pmoB-amoB* and *narB* and negatively correlated with abundance of *phoN* and *phnO*. Moreover, many genes in P cycling (e.g., *phoABR*, *phnAEKLMNI*, *ugpABCQ*, *pstABC*) were positively correlated with the module M00173.

## 4. Discussion

**Figure 7:**
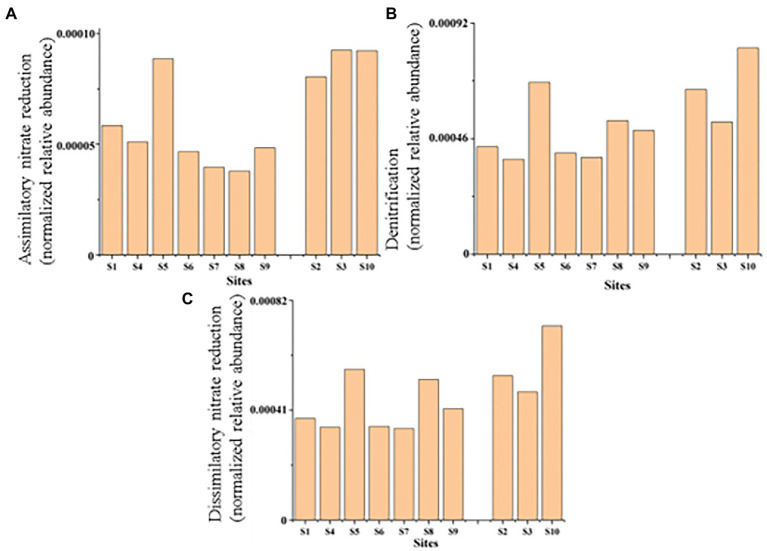
Parts of nitrogen metabolic pathways in sediments of ten typical sites from the Baiyangdian Lake. **(A)** Assimilatory nitrate reduction, **(B)** denitrification, **(C)** dissimilatory nitrate reduction.

Environmental factors are important to determine the composition of microbial communities. Many studies had shown that TN and TP played important roles in composition of bacterial communities (Song et al., 2012; Bergkemper et al., 2016; Wei et al., 2018). In our study, TN and TP greatly affected the bacterial community composition. TN likely showed a relatively greater impact on bacterial communities than TP. We divided the ten points into group 1 (S2, S3, S10) and group 2 (the rest of the samples sites; Figure 7). The values of TN, EC and N/P of group 1 was significantly higher than group 2 (*p* < 0.05). And, there is no obvious difference in total phosphorus content. Compared with the highly contaminated situation (value of TN and TP contents were approximately 4,000 and 800 mg/kg) of Baiyangdian in 2018 reported in January this year (Wang et al., 2022), TN and TP have dropped to around 2,400 and 515 mg/kg, respectively now because of somewhat significant results that the government has achieved in pollution control in recent years. But it is still under eutrophication threat. In addition, in site S3, in the condition of the highest N/P up to 9.04, Proteobacteria had the lowest relative abundance while Chloroflexi had the highest. Also, the function of assimilatory nitrate reduction of N fixation was also the strongest but the functions of C fixation (e.g., reductive citrate cycle, dicarboxylate-hydroxybutyrate cycle, reductive acetyl-CoA pathway) were at an average level. The high N/P might be caused by strong capacity of N removal of Proteobacteria (Nielsen et al., 1999; Ren et al., 2018) and the effects of Chloroflexi in N cycling (Rao et al., 2022), because of the lowest abundance of Proteobacteria and the highest abundance of Chloroflexi. TN and TP were important macronutrients for all biota on earth and were two essential components of the energy metabolism and the genetic backup and stable cell structures. For example, recent researches had provided evidence for that TN was the dominant environmental factor to drive the formation of bacterial community structure and that TP concentration was significantly correlated with the distribution of bacterial communities (Song et al., 2012; Fan et al., 2019). In addition, previous researches showed that microbial community composition could varied with addition of N (Wang et al., 2018). A study showed that the effect of TN on the community structure was mainly caused by pushing the variation of the dominated phyla and influencing the functional microbes of C and N metabolism (Liao et al., 2021). For TN, many studies had shown that the relative abundance of Actinobacteria might be restrained by TN (Pascault et al., 2014; Mao et al., 2019; Ouyang et al., 2020). It was consistent with the community distribution of sample S4, but not the communities of other samples. In addition, at site S4, 3-Hydroxypropionate cycling of C fixation was the strongest while the weakest function of denitrification and complete nitrification of N cycling (might be caused by low abundance of Actinobacteria) which might lead to the high level of TN. TN fixation of sediment might be weaker in areas with high water velocity (Duan et al., 2016). Sample S6, S7, S9 were basically sampled from open water; Sample S1 was sampled from Zhaowangxin river estuary. The relatively low TN content and N/P might be caused by high mobility of water. In addition, sample S10 was sampled from entrance of lake and S2, S3 were, respectively, sampled from the area where was more likely facing pressure of extensive water pollution and eutrophication near to Beihezhuang and Caiputai villages, all of them were relatively severely affected by human activities. The N enrichment in these samples was likely due to the geographic location of the sampling sites. Sample S5 was located near the village but the TN content was relatively low, which suggested that the situation was complicated under field conditions, and there might also be an area with relatively light pollution with less human activities. Sediment pH greatly impacted the TN contents by influencing microbial activities. Microorganisms were most active in neutral conditions while were inhibited in the alkali condition (Bai et al., 2005). Besides, from a global perspective, soil salinity was one of the major environmental factors limiting plant growth and productivity (Huang L. et al., 2017). We found that sediment TN was significantly negatively correlated with pH and positively correlated with EC. It was consistent with the findings of Mei et al. (2014). In lakes, reduction of the external P loading was a prerequisite for water quality improvement. However, lakes showed hardly any signs of recovery after reducing of the external P and it was mainly caused by recycling of P from the P-rich sediments (Lürling and van Oosterhout, 2013). The P cycle genes in Baiyangdian area were relatively rich (Figure 4) indicating that the P cycle in Baiyangdian sediments was relatively strong. This might be explained by the statement that the predominance of organic matter remineralization as the predominant pathway of P cycling in temporally varying bottom water redox conditions (Joshi et al., 2015).

Bacterial community played a key role in ecological functions of sediments. Proteobacteria was reported to contribute to N fixation, photosynthesis, S metabolism, methane metabolism and could degrade proteins, polysaccharides and other organic matter and removed N and P (Liao et al., 2019; Ouyang et al., 2020; Rathour et al., 2020; Duyar et al., 2021). As the dominant colonies in Baiyangdian sediment, the relative abundance of Proteobacteria in bacterial community was significant negatively correlated with Actinobacteria indicating their opposite distribution pattern. The dominance of Proteobacteria in bacterial community and opposite distribution pattern between Proteobacteria and Actinobacteria were also found in other plain lakes with different trophic status (Huang W. et al., 2017), suggesting that a universal mechanism was existed to shape the sediment bacterial community. Following Proteobacteria, Actinobacteria, Chloroflexi and Acidobacteria (Figure 2) were the large phyla within the bacterial community. Actinobacteria produced 70% of the world’s natural antibiotics and had the ability to degrade complex organic compounds (Barka et al., 2016). Recent studies on antibiotics in Baiyangdian showed that there was a real risk of antibiotic contamination in the Baiyangdian area (Zhang et al., 2022). Actinobacteria widely existed in ocean, soil and freshwater ecosystem, and many new species had been found (Wu et al., 2021). Actinobacteria had been identified to be potentially important DDT degraders and had been proved to degrade HCH by producing dechlorinating enzymes (Fudala-Ksiazek et al., 2018; Xu et al., 2021). In Baiyangdian Lake, Hu et al. (2010) had proved that organochlorine pesticides residue existed and HCHs were the predominant contaminants. This could explain why Actinobacteria occupied the second most abundant phylum of bacterial community in Baiyangdian, a typical shallow lake, which was also consistent with other studies (Liao et al., 2019; Ouyang et al., 2020). Then, noticeably, the third most abundance phylum of bacterial community in sediment was Chloroflexi. It was involved in the metabolism of organic matter and the transformation of contaminants (Liao et al., 2021). And, researcher had proved that Chloroflexi were rich in the sediments from eutrophic region (Huang W. et al., 2017).

Except for C metabolism, the biogeochemical processes of N and S also affected the biodegradation in sediments. Microbial groups related to N metabolism, including N_2_ fixation groups and denitrification groups, could improve N availability, because bioavailable N was a major limiting factor in nutrient poor environments (Li et al., 2022). In our study, we found that the relative abundance of each module of the C-N-S cycle was consistent in each sample site, but its absolute abundance was different. It might indicate that in Baiyangdian lake, due to environmental properties, absolute abundance of each module of each sample site was different, but it showed consistent relative strength in the whole area. For examples, the relative abundance of reductive citrate cycle (M00173), denitrification (M00529) and assimilatory sulfate reduction (M00176) were, respectively, highest in C, N, S in all sediment samples. However, assimilatory nitrate reduction, denitrification and dissimilatory nitrate reduction of nitrogen cycling of group 1 were significantly high than group 2 (Figure 7). This indicated that higher N might accelerate the processes of assimilatory nitrate reduction, denitrification and dissimilatory nitrate reduction of nitrogen cycling. In addition, there were also significant differences in genus of *Nocardioides* involved in these pathways. This was consistent with the previous reports that the important effect of *Nocardioides* on nitrogen transformation (Zhang et al., 2021). Microbial processes associated with the S cycle had been extensively studied. Studies had affirmed that the microbial S cycle was among the most active in lakes (Sorokin et al., 2011; Vavourakis et al., 2019). Our research also found that a complete while weaker S cycle between SO_4_^2−^ and HS^−^ might occur in Baiyangdian lake sediments compared to N cycle and C fixation (Figure 4). For N cycle, DNRA had been determined to co-occur with denitrification in estuary sediments (Baker et al., 2015). Also in our study, DNRA co-occurred with denitrification in shallow lake sediment and both of them were relatively active. The most abundant mode of C fixation of Baiyangdian lake was the reductive citrate cycle. It was noted that the reductive acetyl-CoA pathway was found to be most common mode of C fixation and regarded as the response to energy limitation (Hügler and Sievert, 2011). However, in our study, this C fixation pathway was not very strong. As we known, C metabolism pathways were one of the core metabolisms in soil bacterial community (Cai et al., 2020). Carbohydrate metabolism degraded complex organic matter into readily biodegradable substances and provided the necessary energy and matter for growth of bacteria. As a consequence, C fixation and methane metabolism and in Baiyangdian sediment were analyzed to reveal the pathways of organic matter conversion. For C cycle, organic matter was degraded by methanogenic archaea which used a battery of specific enzymes and coenzymes to cut CO_2_ or methyl compounds down and produced CH_4_ by means of biochemical reactions (Cheng et al., 2021). Moreover, in an aerobic state, organic matter could be broken down to produce CO_2_ by microorganisms. Methanogenesis (M00357) in methane metabolism and the reductive citrate cycle (M00173) module in carbon fixation were further analyzed (Figure 6). The highest proportion of M00357 module was the main pathway of methane metabolism. In the methane metabolism M00357 module, acetate was converted to acetyl phosphate via ATP: acetate phosphotransferase (EC 2.7.2.1) again into acetyl-CoA via acetyl-CoA: phosphate acetyltransferase, or to acetyl-CoA via acetate: CoA ligase (EC 6.2.1.1). In addition, acetyl-CoA, coenzyme M-HTP heterodisulfide, 2-(Methylthio) ethanesul-fonate and 5-Methyl-5,6,7,8-tetrahydromethanopterin were interconverted through a series of enzymes and coenzymes. The highest proportion of M00173 module was the main pathway of C fixation. The same was, acetyl-CoA, Citrate and (3S)-Citryl-CoA were interconverted through C fixation process. M00357 module and M00173 module were interconnected by acetyl-CoA. Moreover, many key genes in C, N, P, S cycling were closely related with C fixation. This showed that there was a complex interaction between the metabolic pathways of C fixation and key genes of C, N, P, S biogeochemical cycling. For the P cycle, genes involved in regulation of P starvation (such as *phoB*, *phoR*) were prevalent in soil ecosystem. K01078 (acid phosphatase) and K03788 (acid phosphatase (class B)) belonging to phosphoesterase were not detected in Baiyangdian sediment metagenome, possibly meaning the absence of phosphoesterase metabolism in microbiota. Gene *ppk* was reported to be a phylogenetic marker of phosphorus removal communities and was widely used to investigate P removal organisms. In addition, it was closely linked the patterns underlying endogenous P release from sediments (Lv et al., 2015). So, the dominance of S8 of K00937 (*ppk*) in Baiyangdian sediment metagenome might be associated with the release of underlying endogenous P. The same change trend in the ten sediment samples was also shown in some genes with relatively high abundance, such as, *pstABCS*, *phnCDE* and *ugpABCE*. It might suggest that the strength of P cycling in Baiyangdian lake. And, high microbial potential for efficient phosphate uptake systems (for example, *pstSCAB*) was also detected in P depleted soil (Bergkemper et al., 2016). These genes with high abundance also were screened in Baiyangdian sediments. Hence, the high abundance of these genes in lake sediments might be a common phenomenon. Phosphonoacetate hydrolase (*phnA*) might increase TP content by breaking C-P bonds. This interesting conclusion was also found in our previous studies (Campos et al., 2022). Acid phosphatase *phoN* was involved in the hydrolysis of P and occupied an important position in global P cycling (Rana et al., 2020). Therefore, it might increase the biologically available P by increasing the activity of P-related enzymes, resulting in the decrease of soil P content.

## 5. Conclusion

Baiyangdian lake sediment was under weak alkaline conditions. Compared with the highly contaminated situation of Baiyangdian in 2018, TN content have dropped almost 40% and TP content have dropped about 35%, however it was still under eutrophication threat. TN showed a relatively greater impact on bacterial communities than TP. The status of OPPs was at an acceptable level of risk and was not the main source of TP. Proteobacteria was the most main phylum in sediment, and was proved to indispensable contribute to C, N, P, S cycling. At site S4, 3-Hydroxypropionate cycling of C fixation was the strongest while the weakest function of denitrification and complete nitrification of N cycling. Many genera involved in C, N, S, P cycling processes, such as, *Thiobacillus*, *Nitrosomonas*, *Rhodoplanes* of Proteobacteria; *Nocardioides* of Actinobacteria. *Nocardioides* had played an important role in many metabolic pathways of C, N, P, S cycling. Many key genes in C, N, P, S cycling were closely related to the module reductive citrate cycle. The relative abundance of each module of C-N-P-S cycling was consistent in each sample site, but its absolute abundance was different. However, the abundance of pathways of assimilatory nitrate reduction, denitrification and dissimilatory nitrate reduction of nitrogen cycling in sediments with higher TN content was higher than those with lower TN content. Besides, *Nocardioides* with plural genes encoding key enzymes involved in *nasAB* and *nirBD* gene were involved in these pathways. Carbon metabolism pathways was the core metabolisms in sediment bacterial community. In Baiyangdian lake, the methanogenesis process (involving genes such as *acs*, *hdrA2*…) was the main pathway of methane metabolism and the reductive citrate cycle module (involving genes such as *nifJ*, *oorA*…) accounted for the highest proportion of C fixation processes.

## Data availability statement

The data presented in the study are deposited in the NCBI repository, accession number PRJNA908081.

## Author contributions

BK was responsible for writing manuscript. RX was responsible for writing and correcting manuscript. YH, YW, LZ, and ZW were responsible for measurement of environmental factor. JB, KZ, JA, MJ, and WP were responsible for determining research direction. All authors contributed to the article and approved the submitted version.

## Funding

This study was financially supported by National Natural Science Foundation of China (NSFC) and National Research and Development Agency of Chile(ANID) for the China-Chile Joint Projects on Water Resources Management (grant no. 51961125201 in China and NSFC190012 in Chile), and Fuzhou University Testing Fund (project number: 2022T011).

## Conflict of interest

The authors declare that the research was conducted in the absence of any commercial or financial relationships that could be construed as a potential conflict of interest.

## Publisher’s note

All claims expressed in this article are solely those of the authors and do not necessarily represent those of their affiliated organizations, or those of the publisher, the editors and the reviewers. Any product that may be evaluated in this article, or claim that may be made by its manufacturer, is not guaranteed or endorsed by the publisher.
