# Supplementary material for: Metagenomics reveals biogeochemical processes carried out by sediment microbial communities in a shallow eutrophic freshwater lake
Source: Front Microbiol. 2023 Jan 11;13:1112669. doi: 10.3389/fmicb.2022.1112669 (PMC9874162; doi:10.3389/fmicb.2022.1112669)
Supplement: Supplementary file 1 [file Table_1.docx]

Table S1. Spearman correlation analysis between environmental factors.

|  | **pH** | **TN** | **TP** | **EC** | **SOC** | **N/P** | **OPPs** |
| --- | --- | --- | --- | --- | --- | --- | --- |
| **pH** | 1 | -0.709* | -0.321 | -0.758* | -0.552 | -0.552 | -0.552 |
| **TN** | -0.709* | 1 | 0.091 | 0.842** | 0.770** | 0.794** | 0.406 |
| **TP** | -0.321 | 0.091 | 1 | -0.127 | 0.224 | -0.43 | 0.406 |
| **EC** | -0.758* | 0.842** | -0.127 | 1 | 0.552 | 0.830** | 0.491 |
| **SOC** | -0.552 | 0.770** | 0.224 | 0.552 | 1 | 0.491 | 0.515 |
| **N/P** | -0.552 | 0.794** | -0.43 | 0.830** | 0.491 | 1 | 0.042 |
| **OPPs** | -0.552 | 0.406 | 0.406 | 0.491 | 0.515 | 0.042 | 1 |

* Correlation is significant at the 0.05 level. ** Correlation is significant at the 0.01 level.
