# Supplementary material for: Metagenomics reveals biogeochemical processes carried out by sediment microbial communities in a shallow eutrophic freshwater lake
Source: Front Microbiol. 2023 Jan 11;13:1112669. doi: 10.3389/fmicb.2022.1112669 (PMC9874162; doi:10.3389/fmicb.2022.1112669)
Supplement: Supplementary file 2 [file Table_2.docx]

Table S2 Major taxa involved in key genes of C, N, P, S cycling in Baiyangdian lake sediments based on KEGG annotation results of metagenomes.

| **Cycling processes** | **Metabolic pathways** | **Major taxa** |
| --- | --- | --- |
| **Carbon fixation** | 3-HP/4-HB (*abfD*) | *Rhodoplanes, Syntrophus, Methylibium* |
|  | 3-HP (*mcl, prpE*) | *Nocardioides, Aestuariivirga, Sulfurisoma* |
|  | WL (*acsB, cdhA*) | *Methanothrix, Desulfobacca, Desulfobulbus* |
|  | rTCA (*korA...*) | *Nocardioides, Thiobacillus, Anaeromyxobacter* |
|  | CBB (*prkB, rbcLS*) | *Thiobacillus, Sedimenticola, Sulfuricaulis* |
| **Nitrogen cycling** | *napAB* | *Thiobacillus, Dechloromonas, Ephemera* |
|  | *narGHI* | *Gaiella, Nitrospira, Sinimarinibacterium* |
|  | *nasAB* | *Nocardioides, Dechloromonas, Nocardioides* |
|  | *nifDHK* | *Thiobacillus, Desulfobulbus, Sulfurivermis* |
|  | *nirBD* | *Nocardioides, Sulfuricaulis, Thiobacillus* |
|  | *nirKS* | *Actinomadura, Blastococcus, Gaiella* |
|  | *norBC* | *Thioalkalivibrio, Luteitalea, Cellulomonas* |
|  | *nrfAH* | *Ramlibacter, Anaeromyxobacter, Desulfobulbus* |
| **Sulfur cycling** | *phoD* | *Nocardioides, Blastococcus, Knoellia* |
|  | *ugpQ* | *Nocardioides, Cryobacterium, Solirubrobacter* |
|  | Phytase (*appA...*) | *Solirubrobacter, Nocardioides, Kribbella* |
|  | Phosphonate degradation (*phnP...*) | *Desulfatitalea, Thiobacillus, Candidatus_Methanoperedens* |
|  | Inorganic phosphate solubilizing (*ppa...*) | *Pseudomonas, Nocardioides, Thiobacillus* |
|  | Phosphorus (*phoBRU*) | *Thiobacillus, Nocardioides, Candidatus_Methanoperedens* |
| **Phosphorus cycling** | *aprAB* | *Thiobacillus, Sulfurivermis, Sulfuricaulis* |
|  | *cysNCDHJI* | *Frateuria, Aestuariivirga, Myxococcus* |
|  | *dsrAB* | *Sulfuricaulis, Thiobacillus, Anaeromyxobacter* |
|  | *sat* | *Nocardioides, Thiobacillus, Desulfatirhabdium* |
|  | Sox system (*soxB…*) | *Thiobacillus, Aromatoleum, Sulfuricaulis* |
| **Methanogenesis** | *acs, hdrA2, ackA*…… | *Nocardioides, Gaiella, Anaeromyxobacter……* |
| **Reductive citrate cycle** | *nifJ, oorA, ppdK……* | *Methanospirillum, Candidatus_Methanoperedens, Desulfobulbus……* |
