# Supplementary figures and images for: Metagenomics reveals biogeochemical processes carried out by sediment microbial communities in a shallow eutrophic freshwater lake
Source: Front Microbiol. 2023 Jan 11;13:1112669. doi: 10.3389/fmicb.2022.1112669 (PMC9874162; doi:10.3389/fmicb.2022.1112669)

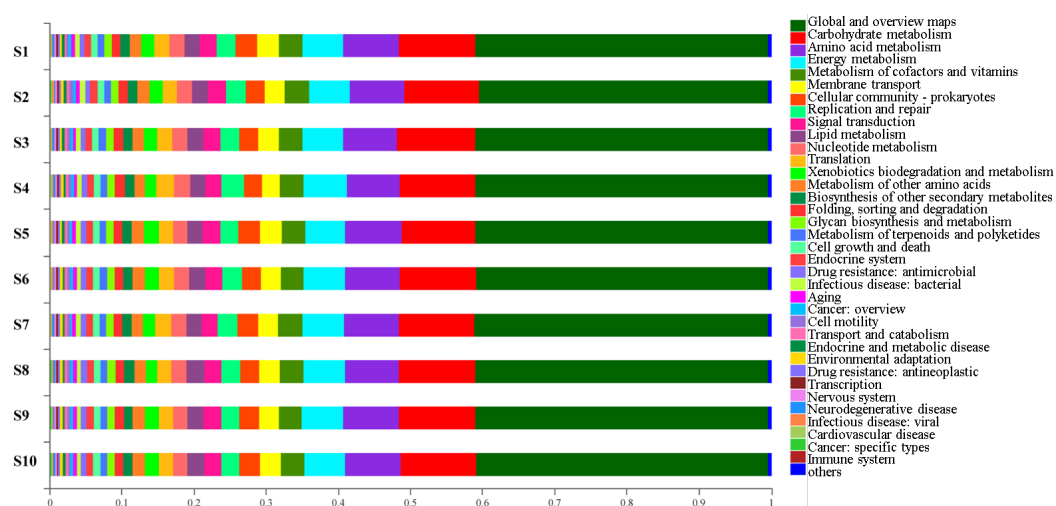

Fig. S1 The composition of metabolic subsystems at KEGG pathway Level 2.

Supplement: Supplementary file 3 [file Image_1.pdf]
